# Composite Graft Reconstruction for Pulmonary Artery Aneurysm Occurring Late After Surgical Pulmonary Valvotomy

**DOI:** 10.7759/cureus.93800

**Published:** 2025-10-03

**Authors:** Yuichi Tatesaka, Tadahito Eda, Satsuki Komoda, Shinichi Mizutani, Hajime Sakurai

**Affiliations:** 1 Cardiovascular Surgery, Okazaki City Hospital, Okazaki, JPN; 2 Cardiovascular Surgery, Japan Community Healthcare Organization Chukyo Hospital, Nagoya, JPN

**Keywords:** composite graft, pulmonary artery aneurysm, pulmonary regurgitation, pulmonary stenosis, rastelli, valvotomy

## Abstract

Pulmonary artery aneurysm (PAA) is considered an uncommon condition, and surgical indications remain unclear. In contrast, pulmonary stenosis (PS) is a common congenital heart disease that often results in pulmonary regurgitation (PR) after intervention and may sometimes require reintervention. In cases of PAA following repair of PS, the caliber mismatch between the dilated pulmonary artery (PA) and the small valve annulus complicates surgery. A composite graft is well-suited to address this issue. A patient underwent surgical valvotomy for PS at age 16. She remained asymptomatic until pregnancy at age 32, when routine cardiac screening was normal; she was then lost to follow-up. At age 72, dyspnea and leg edema led to the diagnosis of combined valvular disease and a 68-mm PAA. Imaging revealed severe PR and right ventricular dysfunction. PA and right ventricular outflow tract reconstruction were performed with a composite graft in a Rastelli-type procedure, along with mitral valve replacement for atrial functional mitral regurgitation and tricuspid annuloplasty for secondary tricuspid regurgitation, in the setting of combined valvular disease complicated by heart failure (HF). The postoperative course was uneventful, and at two years of follow-up, no HF or PA enlargement was observed. This case demonstrates that successful one-stage surgery can be performed in an elderly patient with a PAA and combined valvular disease. A Rastelli-type reconstruction effectively addressed the mismatch between the pulmonary valve and the aneurysmal artery, optimizing hemodynamics and preserving pulmonary flow. Long-term follow-up remains essential to monitor residual tissue and potential late complications.

## Introduction

Pulmonary artery aneurysm (PAA) is rare, with limited opportunities for surgical intervention. The indications for surgery and the operative approach remain unclear [1-3]. In contrast, pulmonary stenosis (PS) is a common congenital heart disease (CHD) that often requires reintervention after repair, with pulmonary regurgitation (PR) being a frequent cause [4,5]. In cases of PAA occurring late after surgery for congenital PS, a caliber mismatch between the pulmonary artery (PA) and the pulmonary annulus may arise. Therefore, in combined surgery for PAA and a small pulmonary valve, this mismatch must be taken into account. In the present case, surgery was performed for heart failure (HF) caused by combined valvular disease, including PR, mitral regurgitation (MR), tricuspid regurgitation (TR), and a coexisting PAA, with PR being a consequence of previously treated PS.

## Case presentation

At age 16, the patient underwent surgical valvotomy under cardiopulmonary bypass for PS, presenting with marked fatigue and exertional dyspnea. Medical records related to the procedure were unavailable due to its antiquity. She remained asymptomatic thereafter. During pregnancy at age 32, routine cardiac evaluation showed no abnormalities, and no follow-up was performed after delivery.

At age 72, the patient experienced progressive fatigue that was initially attributed to aging until the sudden onset of dyspnea and bilateral leg edema prompted emergency evaluation. Chest radiography demonstrated pulmonary congestion, and laboratory testing revealed mildly elevated transaminases. Diuretic therapy improved HF. Subsequent evaluation revealed combined valvular disease, such as MR, PR, and TR, and a PAA.

**Figure 1:**
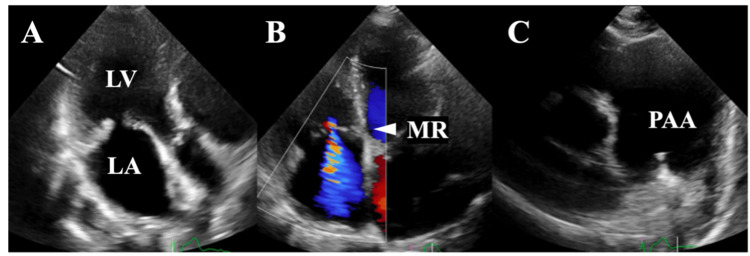
Preoperative transthoracic echocardiography Echocardiography showed moderate to severe MR with tethering of the posterior leaflet (A, B). A PAA was also observed (C). LA: left atrium; LV: left ventricle; MR: mitral regurgitation; PAA: pulmonary artery aneurysm

Transthoracic echocardiography showed moderate to severe MR (regurgitant volume, 32 mL; regurgitant fraction, 50%; vena contracta, 4.6 mm; left ventricular end-diastolic volume index, 27 mL/m²) with posterior leaflet tethering. Moderate PR and TR were also observed (Figure 1).

**Figure 2:**
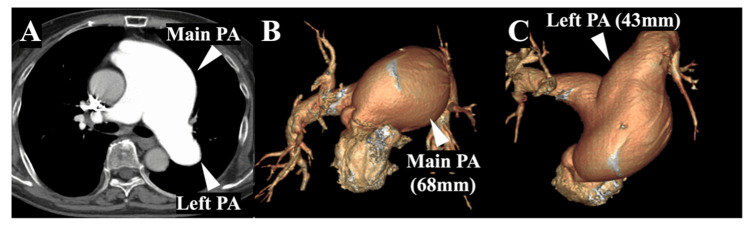
Preoperative CT Contrast-enhanced CT revealed a massively dilated main PA (68 mm) and proximal left PA (43 mm) (A). The PA is shown on 3D CT (B, C). PA: pulmonary artery

CT revealed a massively dilated main PA (68 mm) and proximal left PA (43 mm) with right ventricular and biatrial enlargement (Figure 2).

**Figure 3:**
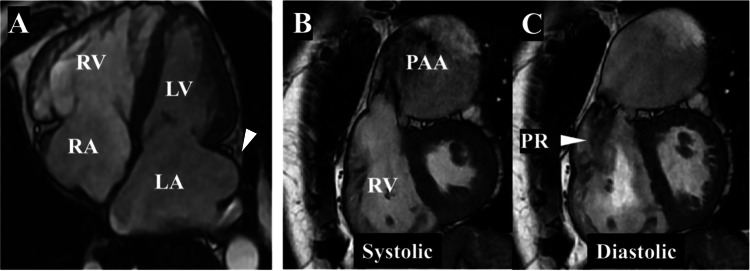
Preoperative MRI MRI revealed biatrial enlargement and right ventricular enlargement. The peripheral portion of the left atrium extended markedly beyond the posterior wall of the left ventricle (A, white arrow). A PAA and PR were also observed (B, C). LA: left atrium; LV: left ventricle; PAA: pulmonary artery aneurysm; PR: pulmonary regurgitation; RA: right atrium; RV: right ventricle

Cardiac MRI confirmed PR with a regurgitant fraction of 40%, a right ventricular ejection fraction of 31%, a right ventricular end-diastolic volume index of 221 mL/m², and a right ventricular end-systolic volume index of 153 mL/m². The left atrial periphery extended markedly beyond the posterior wall of the left ventricle (Figure 3).

Cardiac catheterization demonstrated Sellers grade 4 MR, PA pressure of 39/4 mmHg, pulmonary capillary wedge pressure of 10 mmHg, and left ventricular pressure of 124/0 mmHg. The electrocardiogram demonstrated a sinus rhythm.

Surgical intervention was indicated for HF associated with combined valvular disease and PAA. The PAA met surgical criteria by diameter (discussed below), pulmonary valve replacement was indicated according to the Boston Children’s Hospital criteria [6], and tricuspid/MR met Class IIa (weight of evidence and opinion favored effectiveness and/or usefulness) and Class I (evidence and/or general agreement indicated that the procedure or treatment is effective and/or useful)/IIa criteria, respectively, under the Japanese Circulation Society guidelines. Catheter-based interventions were considered but deemed unsuitable: PR was associated with the PAA, and MR was atrial functional with a short posterior leaflet, precluding durable transcatheter repair. Given the patient’s age but acceptable surgical tolerance, the heart team elected to perform single-stage open surgery to prevent future reinterventions, reduce the risk of PAA rupture or dissection, and enable recovery through rehabilitation.

**Figure 4:**
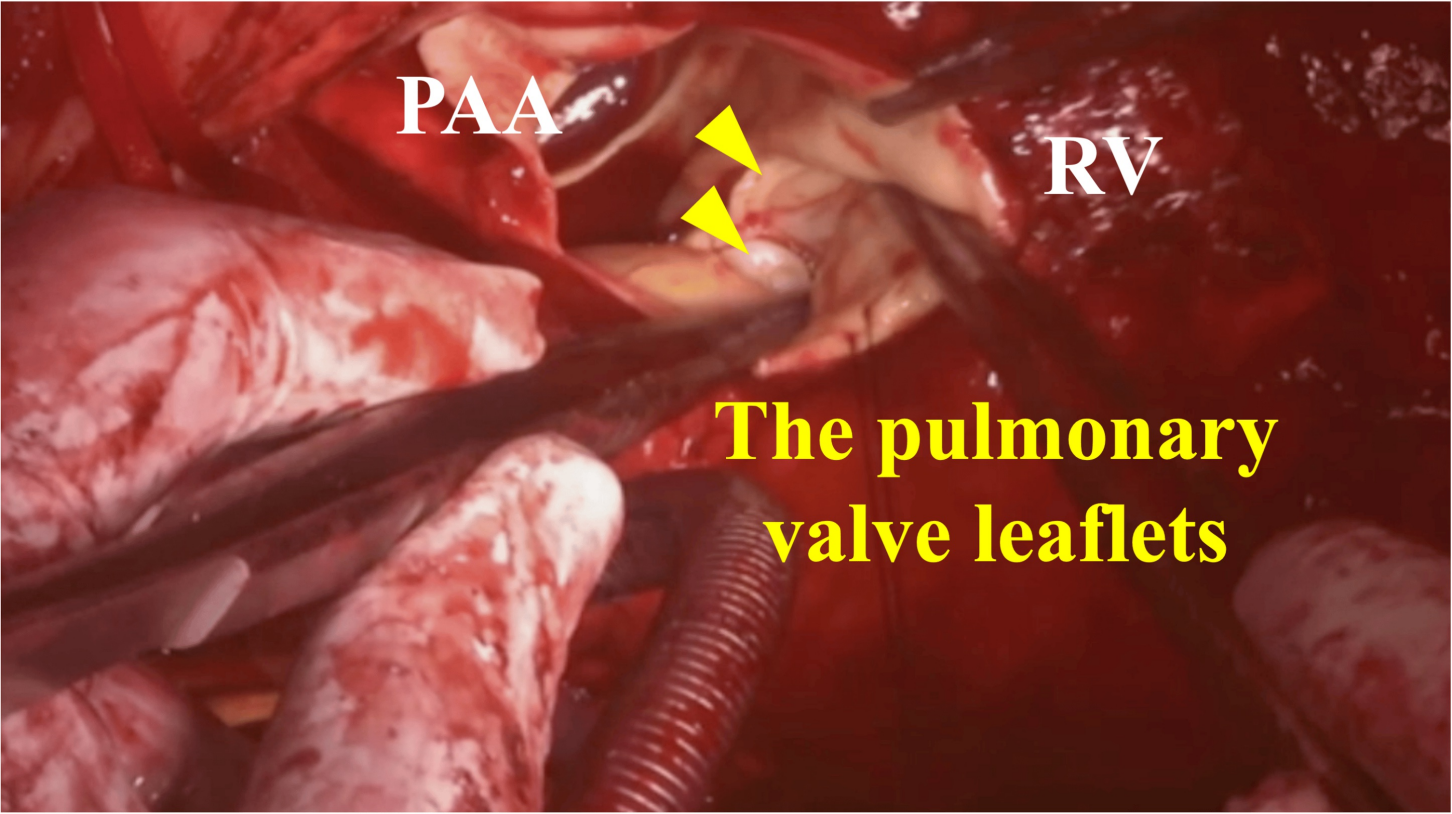
Surgical view of the pulmonary valve The annulus measured 19 mm, and the pulmonary valve leaflets were thickened and shortened (yellow arrow). PAA: pulmonary artery aneurysm; RV: right ventricle

The PAA was incised longitudinally, revealing a pulmonary valve annulus measuring 19 mm (Figure 4).

A 28-mm Triplex-Advanced artificial graft (Terumo Corporation, Tokyo, Japan) was selected to achieve the largest possible conduit while matching the diameters of the left and right pulmonary arteries. The size of the bioprosthetic valve (23-mm INSPIRIS; Edwards Lifesciences, Irvine, CA, USA) was then determined based on the graft diameter, selecting the largest valve that could be accommodated. The dilated proximal left PA was triangularly plicated to achieve an internal diameter of 28 mm. These steps were performed on a beating heart.

After aortic cross-clamping, the mitral valve was addressed first, as adequate exposure could only be obtained under cardioplegic arrest and would have been difficult once the other valves had been implanted. Mitral valve replacement was performed due to atrial functional MR [7], characterized by a tethered posterior leaflet measuring 8 mm, while patch augmentation was avoided to reduce cross-clamp time [8,9]. The tricuspid leaflets were thin and pliable and appeared morphologically normal, but the annulus was dilated, measuring 62 mm from the anteroseptal commissure to the posterior annulus. Tricuspid annuloplasty was then performed using a 28-mm Physio Tricuspid Ring (Edwards Lifesciences).

The pulmonary valve annulus was incised toward the right ventricle, and the previous valvotomy patch was removed. As part of a Rastelli-type reconstruction, the composite graft was first anastomosed to the obliquely cut PA, preserving a 2.5 × 2 cm segment of its posterior and right lateral walls. Preoperative cardiac MRI and intraoperative inspection had confirmed these walls to be thicker and distinct from the aneurysmal portion, suggesting sufficient strength to support bifurcation reconstruction. The graft was subsequently connected to the right ventricle.

**Figure 5:**
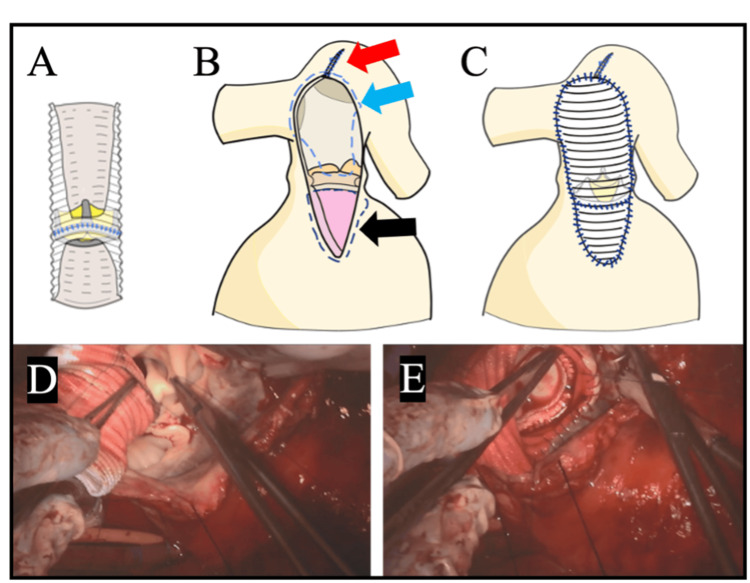
Intraoperative schemas and photographs The composite graft was trimmed (A). The suture line of the PA and RVOT is shown (B). The dilated proximal left PA underwent triangular plication and was sutured (red arrow). The suture line on the PA side is indicated (blue arrow), and the suture line on the cardiac side is indicated (black arrow). Implantation of the composite graft was completed (C). A surgical view shows suturing of the posterior wall on the PA side (D). Another surgical view shows the dorsal aspect of the RVOT, where the graft was sutured to the ventricular myocardium (E). (D) corresponds to part of the blue arrow, and (E) corresponds to part of the black arrow. PA: pulmonary artery; RVOT: right ventricular outflow tract

The right ventricular outflow tract (RVOT) was simultaneously closed with the graft. The fragile right ventricular myocardium required small-bite sutures at appropriate spacing and careful technique. To ensure precision and safety, and taking advantage of preserved left ventricular function, suturing was performed under cardiac arrest. The suture line between the PA and RVOT is shown in Figure 5.

**Figure 6:**
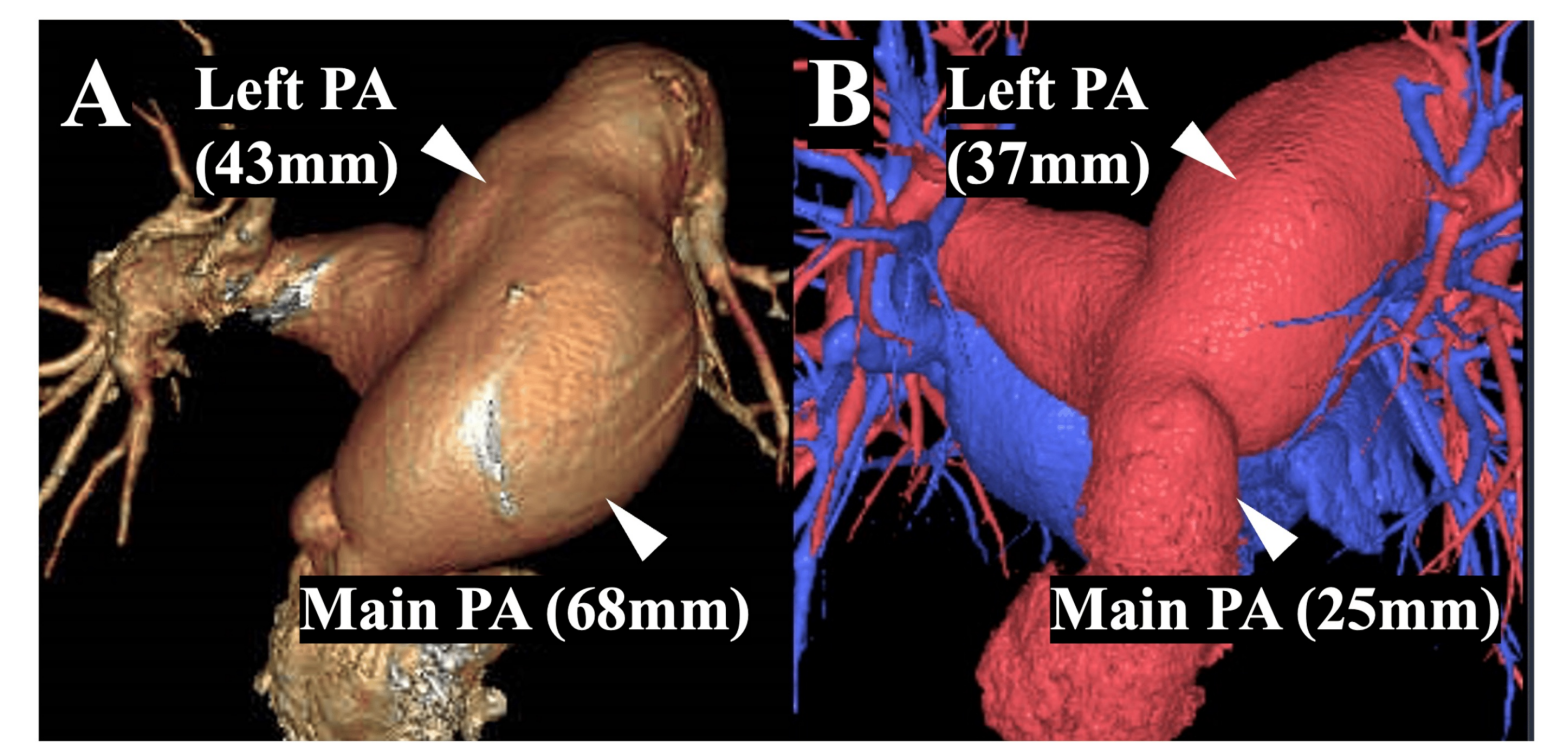
Comparison of 3D CT (A, preoperative; B, two-year postoperative) Preoperative CT showed a massively dilated main PA (68 mm) and a dilated proximal left PA (43 mm) (A). A two-year postoperative CT showed a stable main PA diameter of 25 mm, an unchanged right PA, a reduced caliber of the left PA compared with the preoperative 43 mm, and no graft-related complications (B). PA: pulmonary artery

The postoperative course was uneventful. At two years, the patient remained free of regurgitation and cardiovascular events. Transthoracic echocardiography demonstrated mean gradients of 3 mmHg (mitral) and 6 mmHg (pulmonary), with an estimated right ventricular systolic pressure of 36 mmHg. CT confirmed a stable main PA (25 mm), an unchanged right PA, and a decreased left PA (previously 43 mm), with no graft-related complications (Figure 6).

## Discussion

PAAs may arise from CHD, connective tissue disorders, or iatrogenic causes, with congenital cases generally being larger [10]. Consensus on surgical indications remains elusive [11-13]. Reisenauer et al. classified PAAs ≥8 cm as “giant aneurysms,” recommending proactive surgical intervention; PAAs measuring 5-8 cm require consideration of symptoms, pulmonary hypertension, progressive enlargement, and concomitant cardiac disease; and PAAs ≤5 cm are generally observed [11]. Duijnhouwer et al. proposed three criteria for surgical intervention: (1) CHD as an underlying condition; (2) systolic PA pressure >50 mmHg; and (3) PA diameter >75 mm or an expansion rate ≥2 mm/year [12]. Kreibich et al. suggested surgery for PAAs ≥5.5 cm, with indications including rapid expansion, compression of adjacent structures, thrombus formation, overt symptoms, associated valvular or shunt disease, pulmonary hypertension, or rupture/dissection [13].

In the present case, the PAA measured 68 mm with symptoms, fulfilling the Kreibich criteria (≥55 mm with valvular disease). Although post-stenotic dilatation secondary to PS is possible, serial measurements before and after valvotomy were unavailable, so this could not be confirmed. The aneurysm was therefore presumed congenital. Connective tissue disease was considered unlikely based on age and family history, and no pathological examination was performed. The posterior and right lateral walls (approximately 2.5 × 2 cm) were thick and distinct from the aneurysm, preserving the PA bifurcation. The anterior and left lateral walls were resected because, according to Laplace’s law, wall stress increases with dilatation and thinning. Stress concentration in these regions renders the fragile wall more prone to further dilatation or rupture, whereas the preserved walls maintain sufficient integrity. Regarding the etiology of the PAA, congenital PS and PR were considered contributory, while MR was unlikely to have played a major role, although a minor contribution cannot be entirely excluded.

Isolated PS is a common CHD [14]. Although catheter interventions are now standard [15], surgical valvotomy was the only option 60 years ago [4,5]. Long-term follow-up from the Mayo Clinic showed that 53% of patients required reintervention, with 75% of those cases due to PR [16]. Surgical indications for PR after intervention for PS, frequently based on Boston Children’s Hospital criteria [6], include a regurgitant fraction ≥25%, a right ventricular end-diastolic volume index ≥160 mL/m², a right ventricular end-systolic volume index ≥70 mL/m², a left ventricular end-diastolic volume index ≤65 mL/m², a right ventricular ejection fraction ≤45%, and the presence of moderate TR. The patient in this case met several of these criteria and presented with significant HF symptoms attributable to moderate MR.

Given the marked PA dilatation and combined PR, MR, and TR, a Rastelli-type reconstruction using a composite graft was deemed optimal. While Rastelli-type procedures are commonly performed in pediatric patients with PS, reports of their use in elderly patients with giant PAAs appear to be lacking in the literature. Originally developed for d-transposition of the great arteries with ventricular septal defect and PS, the Rastelli procedure creates an intraventricular baffle directing left ventricular blood to the aorta and reconstructs the RVOT with a conduit. In this case, however, the term “Rastelli-type reconstruction” referred exclusively to reconstruction of the RVOT to the PA. This approach effectively addressed the size mismatch between the small pulmonary valve annulus and the markedly dilated PA. A standard composite graft, such as in the Bentall procedure, would have limited distal PA reconstruction and prosthetic valve implantation. Thus, this technique may have broader applicability in patients with a disease affecting both the pulmonary valve and the PA. In this patient, a bioprosthetic valve was selected to allow for future valve-in-valve interventions and to avoid lifelong anticoagulation.

Regarding prosthetic valve selection, most reports concern bioprosthetic valves in the pulmonary position in pediatric patients; data in older adults are limited, and no reports specifically address composite grafts. Kwak et al. reported a mean age of 14.9 years, with 10-year freedom from reoperation of 77-86% and freedom from prosthetic failure of 84% [17]. Therefore, a bioprosthetic valve was chosen, with the type (pericardial or porcine) left to the surgeon’s preference. Postoperatively, the patient will undergo annual echocardiography and non-contrast CT to monitor prosthetic valve degeneration and distal PA enlargement. Although cardiac MRI could provide a more detailed assessment of vessel wall thickness, it would not alter clinical management; thus, non-contrast CT was deemed sufficient for routine long-term follow-up. Should significant degeneration occur, transcatheter valve-in-valve replacement may be considered for the pulmonary valve, and with further advances in catheter-based therapies, similar interventions may become feasible for the mitral and tricuspid valves as well as the PA itself.

Regarding the mitral valve, the patient was in sinus rhythm with a markedly enlarged left atrium. The posterior leaflet measured only 8 mm, with displacement and tethering consistent with atrial functional MR. As reported by Shibata et al. and Kawamoto et al. [8,9], repair of such short leaflets typically requires complex techniques such as patch augmentation. Such a repair would require extensive enlargement spanning the entire distance between both commissures, which was not feasible given the limited operative time. In addition, given the relatively better long-term outcomes of mitral valve prostheses, mitral valve replacement was chosen to avoid prolonged cross-clamp time.

## Conclusions

The Rastelli-type reconstruction proved effective in this case, addressing the marked size mismatch between a small pulmonary valve annulus and a dilated PAA. This approach optimized pulmonary valve hemodynamics and facilitated surgical repair of the aneurysm. Residual tissue in the distal PA and potential bioprosthetic valve degeneration necessitate careful long-term follow-up, including annual echocardiography and periodic CT to monitor valve function and distal PA status.
